# PTPN21 Overexpression Promotes Osteogenic and Adipogenic Differentiation of Bone Marrow-Derived Mesenchymal Stem Cells but Inhibits the Immunosuppressive Function

**DOI:** 10.1155/2019/4686132

**Published:** 2019-11-21

**Authors:** Huafang Wang, Xiaohang Ye, Haowen Xiao, Ni Zhu, Cong Wei, Xiang Sun, Limengmeng Wang, Binsheng Wang, Xiaohong Yu, Xiaoyu Lai, Shan Fu, He Huang

**Affiliations:** ^1^Bone Marrow Transplantation Center, The First Affiliated Hospital, Zhejiang University School of Medicine, Hangzhou 310003, China; ^2^Department of Hematology, The Sir Run Run Shaw Hospital, Zhejiang University School of Medicine, Hangzhou 310000, China; ^3^Institute of Hematology, Zhejiang University, Hangzhou 310000, China; ^4^Department of Hematology, The First Affiliated Hospital, Zhejiang Chinese Medical University, Hangzhou 310000, China; ^5^Zhejiang Engineering Laboratory for Stem Cell and Immunotherapy, Hangzhou 310000, China; ^6^Zhejiang University-University of Edinburgh Institute, International Campus Zhejiang University, Haining 314400, China

## Abstract

Protein tyrosine phosphatases (PTPs) act as key regulators in various cellular processes such as proliferation, differentiation, and migration. Our previous research demonstrated that non-receptor-typed PTP21 (PTPN21), a member of the PTP family, played a critical role in the proliferation, cell cycle, and chemosensitivity of acute lymphoblastic leukemia cells. However, the role of PTPN21 in the bone marrow microenvironment has not yet been elucidated. In the study, we explored the effects of PTPN21 on human bone marrow-derived mesenchymal stem cells (BM-MSCs) via lentiviral-mediated overexpression and knock-down of PTPN21 in vitro. Overexpressing PTPN21 in BM-MSCs inhibited the proliferation through arresting cell cycle at the G_0_ phase but rendered them a higher osteogenic and adipogenic differentiation potential. In addition, overexpressing PTPN21 in BM-MSCs increased their senescence levels through upregulation of P21 and P53 and dramatically changed the levels of crosstalk with their typical target cells including immunocytes, tumor cells, and vascular endothelial cells. BM-MSCs overexpressing PTPN21 had an impaired immunosuppressive function and an increased capacity of recruiting tumor cells and vascular endothelial cells in a chemotaxis transwell coculture system. Collectively, our data suggested that PTPN21 acted as a pleiotropic factor in modulating the function of human BM-MSCs.

## 1. Introduction

Mesenchymal stem cells (MSCs), found in multiple tissues such as bone marrow and adipose tissue, are a type of multipotent progenitor cell possessing a self-renewal capability and the potential to differentiate into multiple lineages contingent on their exposure to certain soluble factors in their microenvironment, e.g., osteoblasts, adipocytes, and chondrocytes [1–3]. Based on such biology, MSCs have been applied in the clinic for cardiac [4] and musculoskeletal diseases [5]. Meanwhile, their immunosuppressive effects have wider application prospects in graft-versus-host disease (GVHD) [6]. Residing in the bone marrow microenvironment, bone marrow-derived MSCs (BM-MSCs) play a pivotal role in both physiological hematopoiesis and pathological leukemogenesis [7].

Protein tyrosine kinases and protein tyrosine phosphatases (PTPs) are essential regulators of cellular physiological activity and play different roles in regulating tyrosine phosphorylation and the signal transduction pathway [8]. Human tyrosine phosphatase superfamily includes 125 members, collectively referred as PTPome. PTPome can be divided into three groups: the classical PTPs (receptor-typed and non-receptor-typed PTPs), the dual-specificity PTPs, and the low molecular weight PTPs [9]. PTPs participate in fundamental cellular activities, including proliferation, differentiation, and migration. The expression level and activation state of PTPs in MSCs are related to their functional diversity. Receptor-typed PTPQ and receptor-typed PTPF positively regulate the adipogenic differentiation, whereas the expression levels of non-receptor-typed PTP13 and receptor-typed PTP*μ* have a negative correlation with the adipogenic potential of MSCs [10–13]. Meanwhile, non-receptor-typed PTP6 (PTPN6) and non-receptor-typed PTP11 (PTPN11), two src homologous proteins, are demonstrated to positively modulate the osteogenesis of MSCs [14, 15]. Besides, PTPN11 potentially promotes cell migration and reduces cell adhesion through several mechanisms, e.g., promoting focal adhesion kinase phosphorylation [16, 17]. The expression levels of non-receptor-typed PTP2 and PTPN6 are negatively correlated with the proliferation potential of MSCs [18, 19].

Our previous study demonstrated that both de novo and relapsed acute lymphoblastic leukemia (ALL) cells had a higher non-receptor-typed PTP21 (PTPN21) expression level in contrast to the nonmalignant control group and overexpressed PTPN21 in ALL cells effectively promoted their proliferation and drug resistance [20]. Furthermore, our data of whole-exome sequencing suggested that *PTPN21* mutations (exon13: c. 1514C > A: p. P505Q; exon13: c. 1573C > G: p. P525A; exon13: c. 1975 G > A: p. A659T), which were found in two out of thirty cases and disturbed the conserved sequence of PTPN21 protein, were potentially involved in the relapse of ALL [21]. PTPN21 was also reported to control the homeostasis and biomechanics of hematopoietic stem cells [22]. However, the corresponding biological activities of PTPN21 in regulating BM-MSCs have not been reported yet.

In consideration of the complicated function of PTPN21 homologous protein in MSC, we therefore explored the effects of the PTPN21 expression level in regulating proliferation, senescence, osteogenic, and adipogenic differentiation of BM-MSCs. Furthermore, we also investigated the effects of PTPN21 expression in BM-MSCs on the crosstalk activities with their target cells.

## 2. Materials and Methods

### 2.1. Isolation and Culture of BM-MSCs

This study was conducted with full understanding and consent of human subjects and was approved by the Human Ethical Committee of the First Affiliated Hospital of Zhejiang University School of Medicine (approval number 2017-313). Human bone marrow samples were from healthy volunteers, about 2 mL per person. Bone marrow mononuclear cells were separated by density gradient centrifugation and cultured in Dulbecco's modified Eagle's medium, 1 g/L glucose (DMEM, 10-014-CVR, Corning, USA) supplemented with 10% fetal bovine serum (FBS, 10099141C, Gibco, USA). As described in previous research, the medium was replaced after the first 48 h, subsequently replaced once every 72 h. BM-MSCs were passaged when they reached 90% confluence. Passages 3–6 were used in the following experiments.

The human embryonic renal epithelial cell line HEK293T, the human vascular endothelial cell line (ECs), and the human breast cancer cell line MCF7 were purchased from the Cell Bank of Shanghai Institute of Biochemistry and Cell Biology, Chinese Academy of Sciences (Shanghai, China). Both HEK293T cells and ECs were cultured in 4.5 g/L glucose DMEM (10-013-CMR, Corning) supplemented with 10% FBS. MCF7 cells were cultured in a RPMI 1640 medium (10-040-CVR, Corning) supplemented with 10% FBS. All cell lines were cultured at 37°C in a humidified incubator with 5% CO2.

### 2.2. Lentivirus Generation and Transfection

The targeting sequences of PTPN21 5′-ccactgccatttgggttgaaa-3′ and inactive scramble sequences 5′-gttctccgaacgtgtcacgt-3′ were inserted into a pGLV3 lentiviral vector to generate the short hairpin interfering RNA and control plasmids, respectively (GenePharma, China). The human PTPN21 coding sequence (NCBI locus NM_007039.4) with a 3x flag tag attached to the C terminal was cloned into a pGLV3 lentiviral vector to construct the overexpression plasmid. Each plasmid was transferred into HEK293T cells together with lentiviral packaging plasmids pMD2.G (Thermo Fisher Scientific, USA) and psPAX2 (Thermo Fisher Scientific). After 48 h, the viral suspension was collected and filtered with 0.45 *μ*m filter and then directly transfected into BM-MSCs after we removed their original medium. After incubating with viral suspension for 24 h, BM-MSCs were replaced with a fresh medium. The transfection efficiency was evaluated using a fluorescence microscope (Nikon, Japan), RT-qPCR (Quantitative Reverse Transcription PCR), and western blot. The overexpression group was labeled “overexpression,” the knock-down group was labeled “knock-down,” and the control group was labeled “control” in the following.

### 2.3. Cell Counting Kit-8 Assay

Three groups of transfected BM-MSCs including control, overexpression, and knock-down groups (they were abbreviated as three groups of transfected BM-MSCs hereafter) were seeded and cultured in a 96-well flat-bottom plate, at a density of 5 × 10^3^ cells in 100 *μ*L volume per well, with three replicates per group. After 48 h culture, 10 *μ*L Cell Counting Kit-8 (CCK8) reagent (ck04, Dojindo, Japan) was added. After incubation for another 4 h, the OD values at 450 nm were analyzed using a microplate reader (SpectraMax M5, Molecular Devices, USA).

### 2.4. Differentiation of BM-MSCs

Three groups of transfected BM-MSCs were seeded into a six-well plate precoated with 0.1% gelatin overnight (3 × 10^5^ cells/per well). The next day, the induction of differentiation began. For osteogenic differentiation, three groups of cells were induced with an osteogenic differentiation medium (HUXMA-90021, Cyagen, China) that were changed every 3 days. On day 14 of induction, Alizarin Red staining was used to detect the formation of calcium nodules as a measure of osteogenic differentiation. For adipogenic differentiation, adipogenic differentiation medium A (HUXMA-90031, Cyagen) was used for the first 3 days before changing to adipogenic differentiation medium B (HUXMA-90031, Cyagen). After further incubation for 24 h, medium B was discarded and adipogenic differentiation medium A was used once again. After 14 days, Oil red O staining was used to assess the formation of lipid droplets as a measure of adipogenic differentiation. Furthermore, three groups of transfected BM-MSCs were collected to perform RT-qPCR assay on days 0, 7, and 14, and untransfected BM-MSCs were collected for RT-qPCR detection of osteogenic differentiation or adipogenic differentiation on days 0, 3, 7, and 12.

### 2.5. Real-Time Quantitative Polymerase Chain Reaction

Transfected and untransfected BM-MSCs were harvested, and RNA was extracted using the Trizol reagent (15596018, Thermo Fisher Scientific). RNA was reverse transcribed (RT) using a HiScript® Q RT SuperMix for quantitative polymerase chain reaction (qPCR) reagent kit with gDNA Eraser (R223-01, Vazyme, China). The qPCR assay of complementary DNA (cDNA) was performed using AceQ Universal SYBR qPCR Master Mix (Q511-02, Vazyme), with three replicates per reaction. The experiment was performed using the Roche LightCycler® 480 system (Roche Applied Sciences, Switzerland). Results of real-time quantitative PCR were analyzed using the 2^-*ΔΔ*Ct^ method to generate relative expression values. Glyceraldehyde 3-phosphate dehydrogenase (*GAPDH*) was used as the reference gene. The PCR primers used are listed in Table 1.

### 2.6. Western Blot Analysis

Control and overexpression groups of BM-MSCs (1 × 10^6^ cells per sample) were harvested by centrifugation at 300 g for 5 min. The cell pellet was washed twice with 1x PBS and lysed for 1 h in RIPA lysis buffer (P0013C, Beyotime Institute of Biotechnology, China) with protein enzyme inhibitor cocktail (A32963, Thermo Fisher Scientific) in an ice bath. Cell lysates were centrifuged at 12,000 g for 10 min. The supernatant was collected and transferred to another 1.5 mL Eppendorf tube, 5x loading buffer was added to the supernatant, and the mixture was incubated for 10 min at 100°C. Prepared protein samples were loaded onto an SDS-PAGE gel for electrophoresis, after which the gel was transferred to a poly(vinylidene fluoride) membrane. The membrane was blocked in 5% nonfat milk for 1–2 h at room temperature and then incubated with primary antibodies for 6 h followed by HRP-conjugated secondary antibodies (E-AB-1003 and E-AB-1001, Elabscience, USA) for 1 h at room temperature. The specific antibody PTPN21 was from Sigma-Aldrich Technology (SAB1306438, USA). The specific antibodies flag tag (14793), ERK (4695), p-ERK (4370), src (2109), p-src (Tyr416) (6943), CDK4 (12790), CDK6 (13331), P21 (2947), and P53 (2524) were from Cell Signaling Technology, and CCNB1 (A2056), HSP70 antibodies (A10897), and the reference antibody *β*-actin (AC004) were from ABclonal Biotechnology. Immunoreactive bands with electrochemiluminescence (ECL) detection were exposed using a chemiluminescence imaging system (Clinx Science, Shanghai, China).

### 2.7. Senescence Analysis

Three groups of transfected BM-MSCs (2 × 10^4^) were plated into 6-well plates, with three replicates per group. When cells reached 50% confluence, the senescence levels were analyzed by *β*-galactosidase staining (9860, Cell Signaling Technology). After the medium was discarded, cells were fixed with diluted 1x fixative solution for 15 min. The fixative cells were rinsed twice with 1x PBS and then stained with 1 mL staining solution (pH 7). The staining was completed after the final incubation at 37°C in a dry incubator for 24 h. The positively stained cells were observed under a microscope. Five randomly selected fields were used for subsequent counts and statistical analysis.

### 2.8. Flow Cytometry

#### 2.8.1. Cell Cycle Assay

For cell cycle assay, the double Ki67 and 7AAD staining methods were used. Control and overexpression groups of BM-MSCs (2 × 10^5^) were seeded in T25 flasks. After 24 h culture, the cells were collected, fixed, and permeabilized according to the manufacturer's instructions (00-5521-00, Thermo Fisher Scientific). In brief, the collected cells were washed and resuspended in 100 *μ*L 1x staining buffer, then incubated with 5 *μ*L Ki67 antibody (151209, BioLegend, USA) and 10 *μ*L 7AAD (559925, BD Biosciences, USA) at room temperature for 30 min. Samples were analyzed using an FC500 MCL flow cytometer. 20,000 events were collected per sample. The results were analyzed using FlowJo software (FlowJo, Ashland, OR, USA).

#### 2.8.2. Detection of Cell Surface Markers

To detect the surface markers of untransfected and three groups of transfected BM-MSCs, 2 × 10^5^ cells per tube were harvested and incubated with anti-CD73-APC (344005), anti-CD45-APC (368511), anti-CD11b-APC (301309), anti-CD19-PECY7 (302215), anti-CD34-PECY7 (343515), anti-CD90-PE (328109), and anti-CD105-PE (323205) antibodies (all from BioLegend) for 30 min at 4°C. Isotype control antibodies were used to assess nonspecific staining. Staining was detected using an FC500 MCL Flow Cytometer (Beckman Coulter); 20,000 events were collected per sample.

#### 2.8.3. Apoptosis Assay

Three groups of transfected BM-MSCs (2 × 10^5^ cells per tube) were harvested and washed twice with precooled PBS. Then, the cells were resuspended in 100 *μ*L binding buffer that was part of the apoptosis detection kit (559763, BD Biosciences). Cells were incubated with 5 *μ*L Annexin V-PE and 10 *μ*L7-AAD antibodies for 15 min at 4°C. Cells with Annexin V positive/7-AAD negative were defined as early apoptosis, while those Annexin V positive/7-AAD positive were defined as late apoptosis. Staining was detected using an FC500 MCL Flow Cytometer (Beckman Coulter); 20,000 events were collected per sample.

#### 2.8.4. Immunosuppressive Function of BM-MSCs

The method used was similar to that described previously [23]. Three groups of transfected BM-MSCs were seeded and cultured in a 96-well round-bottom plate at different density overnight. Peripheral blood mononuclear cells (PBMCs) from healthy donors were isolated by density gradient centrifugation and subsequently prestained with 5 *μ*M carboxyfluorescein diacetate succinimidyl ester (CFSE, C34554, Thermo Fisher Scientific). Accompanied by stimulation of 2 *μ*g/mL phytohemagglutinin (PHA, L1668, Sigma-Aldrich), the stained PBMCs (5 × 10^4^ cells/well) were cocultured with three groups of transfected BM-MSCs at ratios of 1 : 2.5, 1 : 5, 1 : 10, and 1 : 20, with three replicates per groups. Five days later, PBMCs from different groups were collected and evaluated for cell division. For evaluating regulatory T cell (Treg) induction, T cells were separated from PBMCs by means of magnetic beads, cocultured with three groups of transfected BM-MSCs at a 1 : 10 BM-MSC : T cell ratio for 5 days (2 × 10^5^ T cells/well). Nonadherent cells were collected from the cocultures, and the proportion of Tregs present was evaluated by flow cytometry using the monoclonal antibodies anti-CD25-APC (302609), anti-Foxp3-PE (320107, BioLegend), and anti-CD4-FITC (555346, BD Biosciences). Staining was detected using an FC500 MCL Flow Cytometer (Beckman Coulter); 20,000 events were collected per sample.

### 2.9. RNA Sequencing

Two groups of cells were used for RNA sequencing (RNA-seq): BM-MSC control and BM-MSC overexpressing PTPN21, with three replicates per group. RNA was extracted as described above. The sequencing of Digital Gene Expression Tag Profiling and data analysis were carried out by a commercial company (Huada Biotechnology Co., Wuhan, China). The BGISEQ-500 platform (quantification) was used for RNA-seq libraries, generating a mean of approximately 21.87 M reads per sample. The clean reads were aligned to the reference genome and reference gene with HISAT [24] and Bowtie2 [25], respectively. The RSEM tool [26] was used for the quantification analysis of transcript expression, and the fragments per kilobase million (FPKM) method was used to calculate the expression levels. Absolute log2-based fold change (∣log2 FC∣) ≥ 1 and adjusted *p* value ≤ 0.01 were identified with the NOISeq package as significantly differentially expressed genes (DEGs) between two groups [27]. DAVID database [28] was used to perform Gene Ontology (GO) and Kyoto Encyclopedia of Genes and Genomes (KEGG) pathway enrichment analysis.

### 2.10. Transwell Assay

The migration potential of three groups of BM-MSCs was evaluated using transwell chambers that employ a polycarbonate membrane of 8 *μ*m pore size and 6.5 mm diameter (3422, Corning). The lower compartment of the chamber was loaded with a 600 *μ*L growth medium, while 4 × 10^4^ cells in 200 *μ*L of 1 g/L DMEM without FBS were plated into the upper compartment. After incubating for 24 h, the cells of the upper compartment that had migrated through the membrane and attached to the lower surface were fixed with 4% paraformaldehyde and stained with 4′,6-diamidino-2-phenylindole. Cells were counted in four fields of each well by fluorescence microscopy, and the mean was calculated. To analyze the chemotaxis of ECs and MCF7 cells to different groups of BM-MSCs, 1 × 10^5^ transfected BM-MSCs in 600 *μ*L 1 g/L DMEM without FBS were plated into the lower compartment to settle overnight. The next day, 4 × 10^4^ ECs or MCF7 cells in a 200 *μ*L basic medium without FBS were seeded into the upper compartment. Subsequent operations were the same as described above.

### 2.11. Statistical Analysis

GraphPad Prism 5.0 software was used to perform statistical analysis. Error bars indicate the standard deviation of each group of data. Comparisons between two groups were carried out using a two-sided *t*-test. A *p* value < 0.05 was considered statistically significant. *p* < 0.05 was indicated by ^∗^, *p* < 0.01 as ^∗∗^, and *p* < 0.001 as ^∗∗∗^.

## 3. Results

### 3.1. Characterization of BM-MSCs

**Figure 1:**
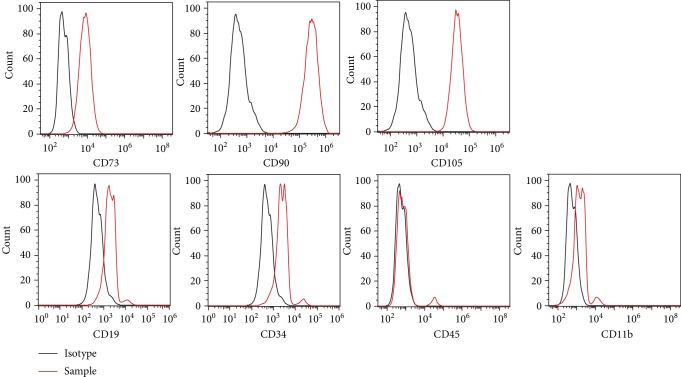
Phenotypes of BM-MSCs. Flow cytometry analysis of cell surface markers of untransfected BM-MSCs. The black-lined histograms indicate isotypic controls.

**Figure 2:**
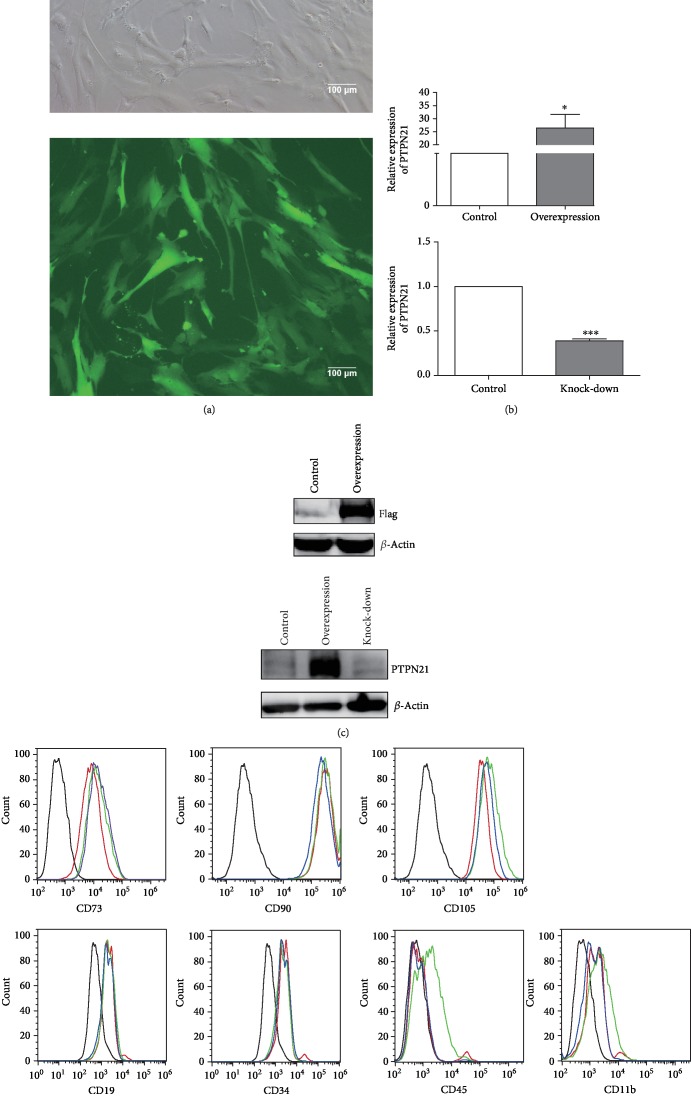
Phenotypes of different groups of transfected BM-MSCs. (a) The transfection efficiency of the control group is shown. No less than 90% of BM-MSCs expressed green fluorescent protein. Scale bar = 100 *μ*m. The efficiency of overexpression or knock-down of PTPN21 was confirmed by RT-qPCR (b) and western blot analysis (c). A flag tag was attached to the C terminus of *PTPN21* coding sequences in the overexpression plasmid, which acted as a reporter gene for transfection efficiency identification. (d) Flow cytometry analysis of cell surface marker expression by transfected BM-MSCs. The black-lined histograms indicate isotypic controls, while the red-lined, blue-lined, and green-lined histograms represent the control, overexpression, and knock-down groups, respectively. All experiments were repeated at least three times. Data are presented as mean ± SD. ^∗^*p* < 0.05.

BM-MSCs were analyzed by flow cytometry as described in previous reports [23, 29]. The cells were positive for the surface markers CD73, CD90, and CD105 but negative or slightly positive for CD34, CD45, CD11b, and CD19 (Figure 1). We generated BM-MSCs with PTPN21 overexpression or knock-down by lentiviral transfection, and the efficiency of PTPN21 overexpression or knock-down in BM-MSCs was confirmed by RT-qPCR and western blot assays. RT-qPCR showed that the expression level of PTPN21 in the overexpression group has an average 25-fold increase compared with the control group and that in the knock-down group had an average of 63% decrease (Figures 2(a)–2(c)). BM-MSCs with PTPN21 overexpression or knock-down displayed similar spindle-shaped morphology and immune-phenotype compared with wild-type cells (Figure 2(d)).

### 3.2. The Overexpression of PTPN21 Inhibited the Proliferation through Preventing BM-MSCs Entering Cycling State

**Figure 3:**
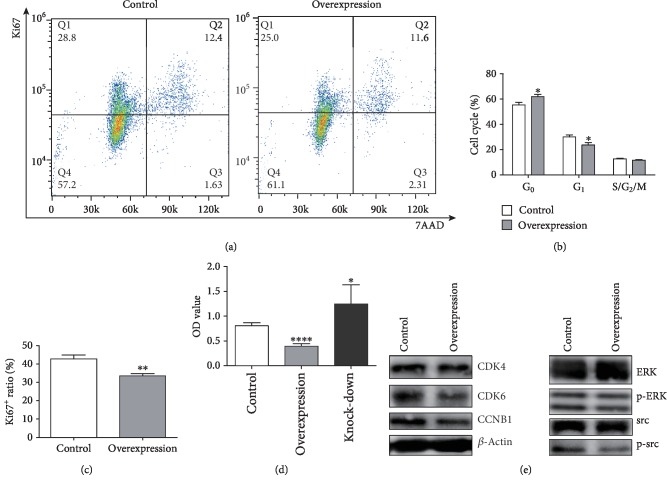
Cellular proliferation and cell cycle in BM-MSCs. (a–c) Typical flow cytometric results of cell cycle and proliferation analysis in the different groups of BM-MSCs. (a) The quadrant with Ki67^−^7AAD^−^ represents the G_0_ phase, Ki67^+^7AAD^−^ represents the G_1_ phase, and Ki67^+^7AAD^+^ represents the S/G_2_/M phase. (d) Statistical analysis of the OD values of three different groups of BM-MSCs from the CCK8 assay. (e) Western blot analysis of some proliferation and cell cycle-associated molecules in BM-MSC control and overexpression groups. All experiments were repeated at least three times. Data are presented as mean ± SD. ^∗^*p* < 0.05, ^∗∗^*p* < 0.01, and ^∗∗∗^*p* < 0.001. ns: no significance.

We compared the cell cycle distribution of control and PTPN21 overexpression groups. The overexpression group had a decreased number of cells in the G_1_ phase (22% ± 0.62% vs. 30.1% ± 1.31%) and a maintained increased number of cells in the G_0_ phase (63.37% ± 0.49% vs. 55.4% ± 1.77%) as compared with the control group (Figures 3(a)–3(c)). In addition, the proliferation levels at 48 h in all three groups were also analyzed. The overexpression group had a lower absorbance than the control group (0.39 ± 0.05 vs. 0.80 ± 0.06), and consistent with this result, the PTPN21 knock-down group had a relatively higher absorbance (1.25 ± 0.35 vs. 0.80 ± 0.06) (Figure 3(d)). We then evaluated the protein expression levels of several proliferation-associated molecules in BM-MSCs with PTPN21 overexpression. The overexpression group had lower expression of p-src, p-ERK, CDK4, CDK6, and CCNB1 molecules (Figure 3(e)).

### 3.3. The Overexpression of PTPN21 Promoted the Osteogenic and Adipogenic Differentiation of BM-MSCs

**Figure 4:**
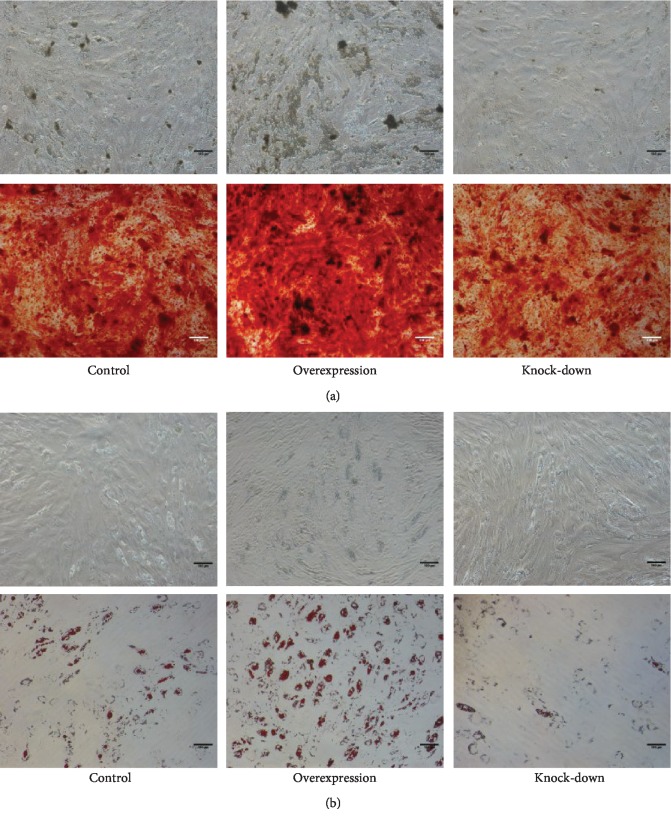
The osteogenic and adipogenic differentiation potential of different groups of BM-MSCs. (a) Alizarin Red staining was used to detect the osteogenic differentiation of three groups of BM-MSCs, and the density of calcified deposits was used to evaluate the level of osteogenic differentiation. Scale bar = 100 *μ*m. (b) Oil red O staining was used to evaluate adipogenic differentiation of three groups of BM-MSCs, and the area of lipid droplets was used to measure the level of adipogenic differentiation. All experiments were repeated at least three times. Scale bar = 100 *μ*m.

**Figure 5:**
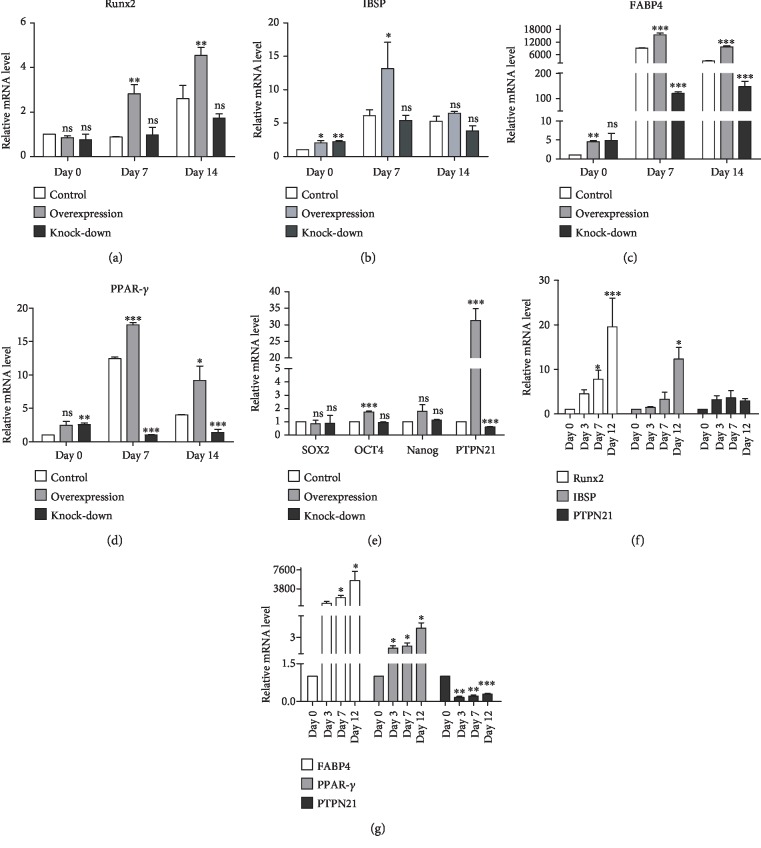
Statistical analysis of osteogenic and adipogenic differentiation data. (a–d) Statistical analysis of mRNA expression of the osteogenic genes *RUNX2* and *IBSP* and the adipogenic genes *FABP4* and *PPAR-γ* on days 7 and 14 of induction in three groups of BM-MSCs. (e) The mRNA expression levels of pluripotency-associated genes *Nanog*, *OCT4*, and *SOX2* in the different groups. (f) mRNA expression of *RUNX2*, *IBSP*, and *PTPN21* during osteogenic differentiation of untransfected BM-MSCs. (g) mRNA expression of *PPAR-γ*, *FABP4*, and *PTPN21* during adipogenic differentiation of untransfected BM-MSCs. All experiments were repeated at least three times. Data are presented as mean ± SD. ^∗^*p* < 0.05, ^∗∗^*p* < 0.01, and ^∗∗∗^*p* < 0.001. ns: no significance.

We further assessed the potential of three groups of transfected BM-MSCs to differentiate towards osteogenic and adipogenic lineages in vitro. Compared with the control group, the overexpression group displayed greater osteogenic and adipogenic differentiation capacity after induction for 14 days, as shown by Alizarin Red staining and Oil red O staining. Correspondingly, both osteogenic and adipogenic differentiation levels were lower in the knock-down group (Figures 4(a) and 4(b)). Besides, the expression levels of specific osteogenic and adipogenic markers were also analyzed by RT-qPCR. Based on these staining results, the expression levels of both the osteogenic markers, *RUNX2* and *IBSP*, and the adipogenic markers, *PPAR-γ* and *FABP4*, were higher in the overexpression group and lower in the knock-down group as compared with the control group at the induction time of 7 and 14 days (Figures 5(a)–5(d)). To assess the effects of PTPN21 regulating the stemness of BM-MSCs, the expression of the pluripotency-associated genes *NANOG*, *SOX2*, and *OCT4* was measured via RT-qPCR. The overexpression of PTPN21 significantly upregulated the expression level of *OCT4* compared with the control group; the expression of *NANOG* had an increased trend, although significant difference was unreached (Figure 5(e)). To analyze the expression levels of PTPN21 during the osteogenic and adipogenic differentiation of BM-MSCs, we examined the endogenous mRNA expression of *PTPN21* on days 0, 3, 7, and 12 of induction. Compared with undifferentiated BM-MSCs on day 0, the expression level of *PTPN21* increased during osteogenic differentiation without reaching statistical significance, while significantly decreased during adipogenic differentiation (Figures 5(f) and 5(g)).

### 3.4. Overexpression of PTPN21 Impaired the Immunosuppression Potential of BM-MSCs

**Figure 6:**
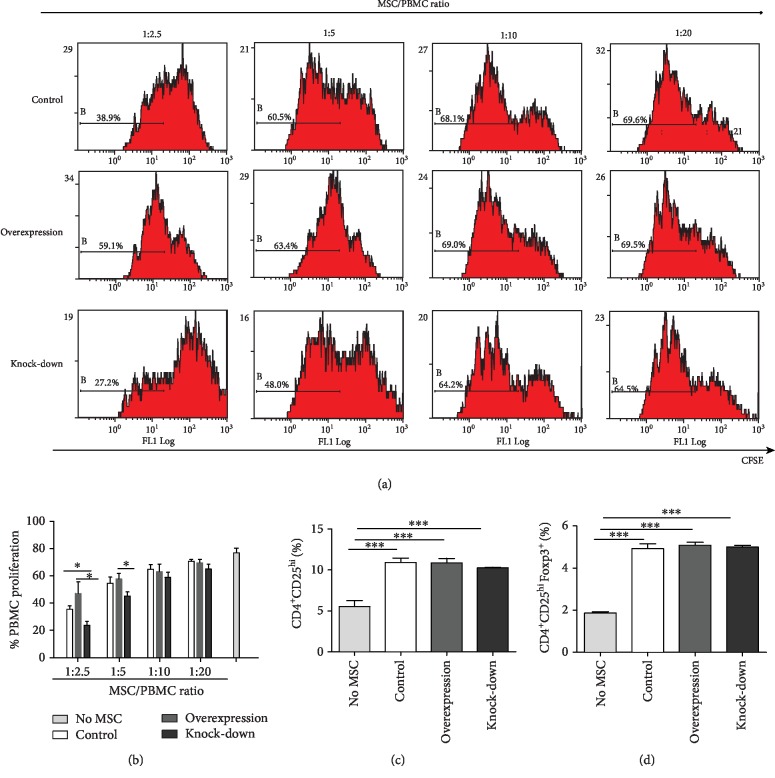
Immunosuppressive functions of BM-MSCs. (a–b) The result of flow cytometry analysis showing the effect of three groups of BM-MSCs on the proliferation of PHA-activated PBMCs. PBMCs were cocultured with different ratios of BM-MSCs for 5 days. (c–d) The proportion of CD4^+^CD25^high^ cells and CD4^+^CD25^+^Foxp3^+^ cells in the gate of CD4^+^ cells. All experiments were repeated at least three times. Data are presented as mean ± SD. ^∗^*p* < 0.05, ^∗∗∗^*p* < 0.001.

Three groups of BM-MSCs were cocultured with PHA-activated PBMCs, and the proliferation-inhibition effect of the PBMCs was used to evaluate the immunosuppressive capacity of the BM-MSCs. Each group of BM-MSCs was mixed with healthy donor-derived PBMCs at four ratios (1 : 2.5, 1 : 5, 1 : 10, and 1 : 20). Compared with the control group, the proliferation level of PBMCs cocultured with BM-MSCs overexpressing PTPN21 was significantly increased in a dose-dependent manner. Correspondingly, that in BM-MSCs with the PTPN21 knock-down group was decreased accordingly (Figures 6(a) and 6(b)).

Moreover, we evaluated the capacity of BM-MSCs with PTPN21 overexpression or knock-down to induce purified T cells to transform into Tregs. After coculturing with T cells for 5 days, the percentages of CD4^+^CD25^high^ cells and CD4^+^CD25^+^Foxp3^+^ cells were significantly increased in control (10.91% ± 0.43%, 4.92% ± 0.19%), overexpression (10.87% ± 0.45%, 5.09% ± 0.12%), and knock-down (10.26% ± 0.07%, 5.0% ± 0.07%) groups, compared with control T cells without being cocultured with BM-MSCs (5.53% ± 0.06%, 1.86% ± 0.06%), whereas it has no significant difference among the three types of cocultured groups (Figures 6(c) and 6(d)).

### 3.5. Overexpression of PTPN21 Accelerated the Senescence of BM-MSCs

**Figure 7:**
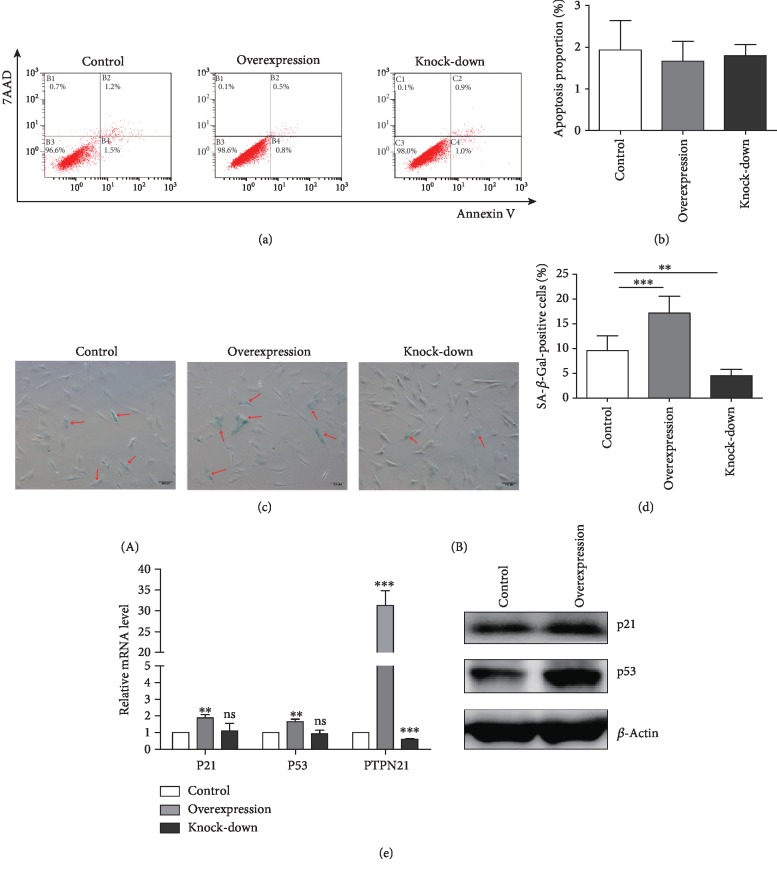
Apoptosis and senescence in BM-MSCs. (a) Flow cytometry analysis of apoptosis in three transfected groups. (b) Statistical analysis of total apoptotic cells in the three groups. (c) SA-*β*-Gal staining in the different groups. SA-*β*-Gal-positive cells are indicated by red arrows. Scale bar = 100 *μ*m. (d) Statistical analysis of SA-*β*-Gal-positive cells in the different groups. (e) mRNA expression (A) and protein expression (B) of *p53*, *p21*, and *PTPN21* in the three groups. All experiments were repeated at least three times. Data are presented as mean ± SD. ^∗∗^*p* < 0.01, ^∗∗∗^*p* < 0.001. ns: no significance.

To evaluate the effect of PTPN21 on apoptosis of BM-MSCs, the apoptosis proportion of three groups of transfected cells was analyzed by Annexin V-FITC/PI staining. There was no significant difference among the overexpression (2.37% ± 0.82%), knock-down (2.6% ± 0.59%), and control (2.77% ± 0.12%) groups (Figures 7(a) and 7(b)). Senescence-associated *β*-galactosidase (SA-*β*-Gal) was used as a marker to analyze the senescence percentage of three groups. The proportion of SA-*β*-Gal-positive cells in the overexpression group (17.14% ± 3.05%) was significantly higher than that in the control group (9.58% ± 2.81%) or the knock-down group (4.51% ± 1.16%) (Figures 7(c) and 7(d)). We also assessed the expression of the senescence-associated markers P21 and P53. Concordant with the results of SA-*β*-Gal staining, the overexpression group had a higher mRNA and protein expression level of *P21* and *P53* than the control group, while there was no significant difference between the knock-down group and the control group (Figure 7(e)).

### 3.6. Gene Profiling Analysis Showed the DEGs upon PTPN21 Overexpression

**Figure 8:**
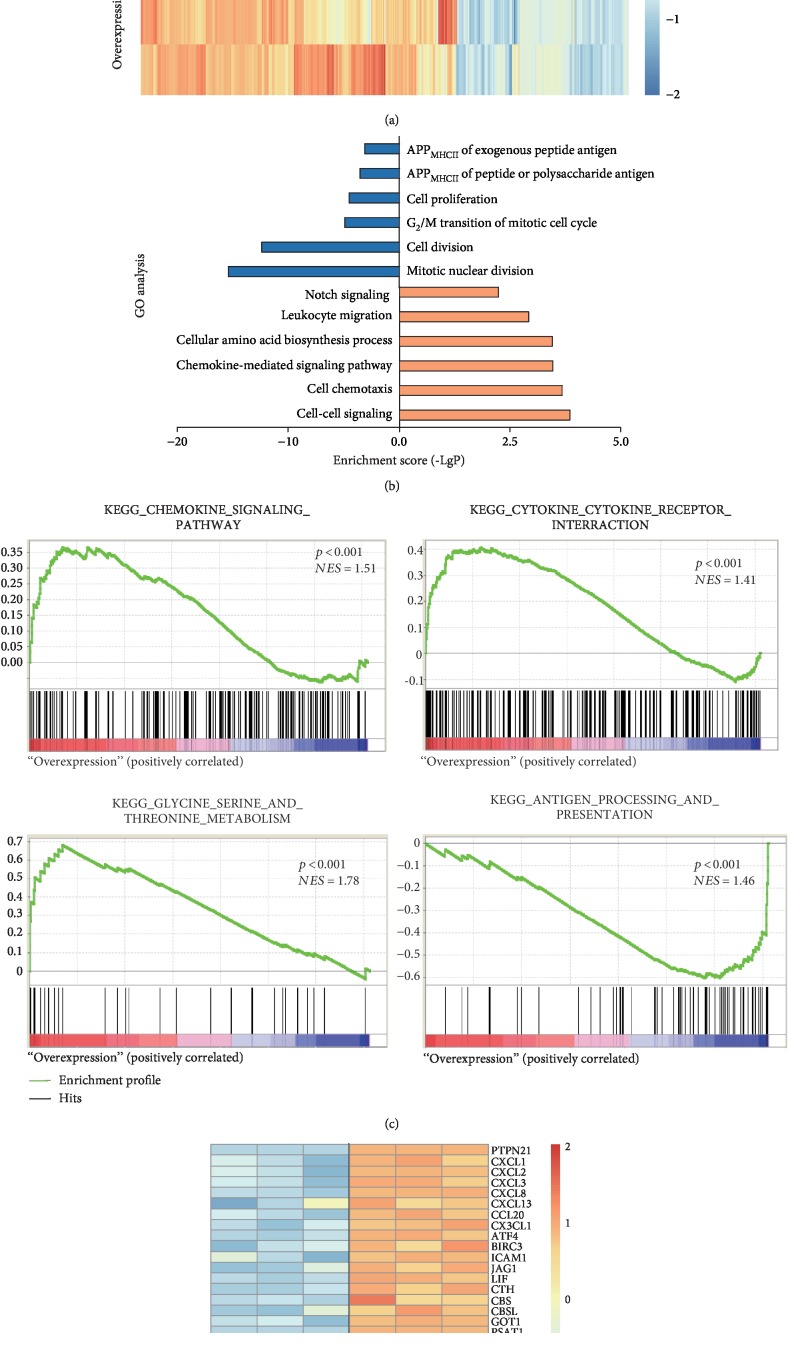
Analysis of transcriptome data in the control and overexpression BM-MSCs. (a) Heat map of DEG cluster. The expression levels of 435 genes change significantly in the overexpression group compared to the control group (*p* ≤ 0.001, (∣log2 FC∣) ≥ 1). Of these, 282 are commonly upregulated and 153 are commonly downregulated. (b) GO analysis of DEGs. Selected significant ontology terms are shown. The blue-colored columns indicate downregulation genes, while the orange-colored columns indicate upregulation genes. (c) GSEA analyses of gene expression profile in overexpression versus control cells. The normalized enrichment score (NES) and *p* values are shown. (d) Heat map of enriched DEGs between two groups is shown. APP_MHCII_: antigen processing and presentation via MHC class II.

To explore the underlying mechanisms of PTPN21 regulating BM-MSCs phenotypes such as the moderative cellular proliferation and increased senescence, RNA-seq assay was performed in BM-MSCs with PTPN21 overexpression or control cells, respectively. Cluster software and Euclidean distance matrix were used for the hierarchical clustering analysis of the expressed genes and sample program at the same time (Figure 8(a)). In the screened DEGs, the overexpression group had 282 upregulated genes and 153 downregulated genes compared with the control group. Gene Ontology (GO) analysis demonstrated that the upregulated DEGs were mainly enriched in the cell-cell signaling pathway, cell chemotaxis, cytokine-cytokine receptor interaction, cellular amino acid metabolism, leukocyte migration, and Notch signaling molecules. The downregulated DEGs were mainly enriched in cell mitotic division, cell cycle, cellar proliferation, and antigen processing and presentation (Figure 8(b)). KEGG pathway enrichment analysis showed the terms of ranking top five upregulated pathways and downregulated pathways, respectively (supplemental [Supplementary-material supplementary-material-1]). Moreover, gene set enrichment analysis (GSEA) of gene expression profile revealed the similar conclusion: there was significant enrichment in the chemokine signaling pathway, cytokine-cytokine receptor interaction, glycine-serine-threonine metabolism, and antigen processing and presentation pathway (Figure 8(c)). The DEGs of the most distinct were screened to depict heat map, including signaling pathway-associated molecules: *CXCL1*, *CXCL2*, *CXCL3*, *CXCL8*, *CXCL13*, *CCL20*, and *CX3CL1*; TNF signaling-associated molecules: *ATF4*, *BIRC3*, *ICAM1*, *JAG1*, and *LIF*; amino acid metabolism-associated molecules: *CTH*, *CBSL*, *CBS*, *GOT1*, and *PSAT1*; cell cycle-associated molecules: *CCNB1*, *CDK1*, and *TUBA4A*; cell proliferation-associated molecules: *AURKB*, *BHLHE41*, and *FSCN1*; and antigen processing and presentation-associated molecules: *HSPA1A*, *HSPA1B*, *HSPA2*, *HLA-DMB*, and *HLA-DRA* (Figure 8(d)).

To validate the results of RNA-seq, RT-qPCR was used to assess the expression of the genes shown in Figure 8(c) in the control and overexpression groups. *HSPA1*, *HLA-DME*, *HLA-DQB1*, *AURKB*, *CHST4*, *GDF5*, and *LIF* were comparably expressed in the control and overexpression groups. The others were identical to the RNA-seq result. Meanwhile, chemokine-associated receptors *CXCR1*, *CXCR2*, *CXCR3*, *CXCR4*, *CXCR5*, and *CCR6* were also detected. Except for the increased expression of *CXCR3* in the overexpression group, other molecules did not differ significantly between the two groups (supplemental [Supplementary-material supplementary-material-1]).

### 3.7. Overexpression of PTPN21 in BM-MSCs Promotes the Recruitment of ECs and MCF7 Cells

**Figure 9:**
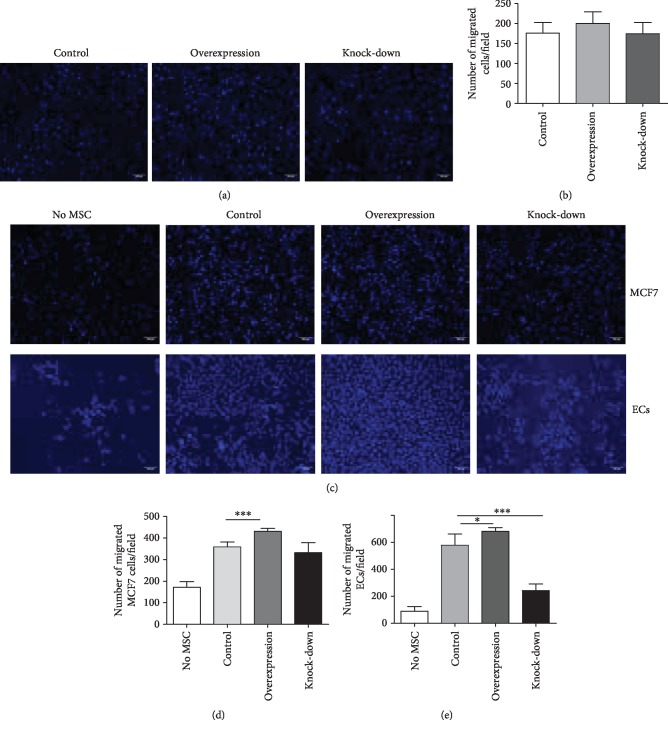
Migration potential and recruitment capability of different groups of BM-MSCs. (a–b) Migration level of different groups of BM-MSCs assessed by transwell assay. Scale bar = 100 *μ*m. (c–e) Migration level of MCF7 cells and ECs cocultured with different groups of BM-MSCs in a chemotaxis transwell system. All experiments were repeated at least three times. Data are presented as mean ± SD. ^∗^*p* < 0.05, ^∗∗∗^*p* < 0.001. Scale bar = 100 *μ*m.

The transwell assay was applied to assess the migration capacity of different groups of BM-MSCs. The number of migration cells had no significant difference among the control (176.17 ± 24.56/field), overexpression (200 ± 26.97/field), and knock-down (174.43 ± 26.52/field) groups (Figures 9(a) and 9(b)). RNA-seq data showed BM-MSCs overexpressing PTPN21 had a higher expression of chemokines, and we therefore evaluated their recruitment capability to MCF7 cells and ECs, both of which highly expressed specific chemokine receptors [30, 31]. Consistent with the RNA-seq data, both MCF7 cells and ECs had a higher migration number in the PTPN21-upregulated BM-MSC group compared to the control group (430.33 ± 12.66/field vs. 358.83 ± 21.46/field, 681.67 ± 26.45/field vs. 579.6 ± 73.75/field) (Figures 9(c)–9(e)).

## 4. Discussion

In this study, we investigated the effect and mechanism of PTPN21 regulating the biological function of BM-MSCs, using lentiviral-mediated overexpression and knock-down of PTPN21. The results demonstrated that overexpression of PTPN21 reduced the short-term proliferation rate of BM-MSCs, and cell cycle analysis showed that overexpressed PTPN21 in BM-MSCs arrested cell cycle at the G_0_ phase accordingly. We then studied the signaling pathways involved in PTPN21 upregulation. It was speculated that overexpressed PTPN21 in BM-MSCs inhibited the MAPK signaling pathway, leading to an impairment in the proliferation ability. Moreover, downregulation of cell cycle-associated proteins CDK4 and CDK6 was also detected, which potentially contributed to the inhibition of cell cycle progression. The study indicated that overexpressing PTPN21 in BM-MSCs inhibited the proliferation capacity partially through preventing them entering cycling state.

Our results revealed that overexpressed PTPN21 in BM-MSCs promoted both adipogenesis and osteogenesis. In addition, the role of PTPN21 in 3T3-L1 preadipocytes was also assessed. 3T3-L1 cells were induced to differentiate into mature adipocytes, using a protocol described previously [13]. Again, overexpressing PTPN21 promoted adipogenesis of 3T3-L1 cells (supplemental [Supplementary-material supplementary-material-1]). Meanwhile, we found PTPN21-overexpressed BM-MSCs had a higher expression of *OCT4* and *Nanog*, although the latter did not reach statistical significance. Nanog, SOX2, and OCT4 are reported to maintain the pluripotent properties, not only of pluripotent stem cells but also of adult stem cells such as MSCs [23]. Ectopic expression of OCT4 improved adipogenesis, and Nanog and OCT4 overexpression promoted chondrogenesis in BM-MSCs [32]. The latest study also reported that the expression levels of *OCT4* and *Nanog* were also positively related to osteogenesis during osteogenic differentiation of human amniotic-derived MSCs [33]. The result suggested that upregulation of PTPN21 in BM-MSCs might better maintain their pluripotency and increase their differentiation potential, which was consistent with the latest report that PTPN21 played an essential role in the homeostasis of hematopoietic stem cells [22]. However, there seem to exist controversy between the lower proliferation and higher differentiation potential in PTPN21-overexpressed BM-MSCs. In our study, three groups of transfected BM-MSCs were in an overconfluence state and had a similar cell number when the induction of differentiation began. Therefore, the effects of PTPN21 on the proliferation of BM-MSCs could be almost neglected in the differentiation assays, and upregulation of PTPN21 was able to accelerate the process of differentiation under the inducing condition. Besides, previous studies reported that glucocorticoid-induced leucine zipper affected the differentiation potential independent of the regulation of proliferation capacity [34] and PTPN6 promoted the osteogenesis [15] but negatively regulated the proliferation level in MSCs [18]. Likewise, we speculate that PTPN21 regulated the osteogenic and adipogenic differentiation of BM-MSCs independent of regulation of proliferation.

BM-MSCs, an essential component of the bone marrow microenvironment, are involved in processes such as hematopoiesis and tumorigenesis through crosstalk with their target cells [35, 36]. Under normal conditions, BM-MSCs have a remarkable capacity of inhibiting the immune response, which is known as immunosuppression [37]. The immunosuppressive activities include releasing immunosuppressive molecules, inhibiting the proliferation of B and T cells, and inducing the generation of Treg cells, the activation of NK cells, and the maturation of dendritic cells. Based on the characteristic, BM-MSCs have been used to prevent graft-versus-host disease and treat autoimmune diseases such as inflammatory bowel disease (IBD) [38]. In our study, despite the proliferation of PHA-activated PBMCs which was suppressed by BM-MSCs of all three transfected groups in a dose-dependent manner, this suppressive effect was significantly impaired in the PTPN21-overexpression group. Another route which BM-MSCs modulate immune response is to induce Tregs [39–41], via an underlying mechanism involving cell contact and soluble factors, e.g., TGF-*β*1 and PGE2 [23, 40, 42]. Our results revealed no marked effects on the number of Treg induced by PTPN21 overexpressing MSCs, which indicated that PTPN21 participated in immunosuppression independent of its immunomodulatory effect on Tregs in BM-MSCs. Further exploration found overexpression of PTPN21 had no direct effects on apoptosis of BM-MSCs, while the detection of SA-*β*-Gal and senescence-associated genes revealed an increase in the incidence of senescence in PTPN21-upregulated BM-MSCs, which potentially impaired their immunosuppression capacity. Our results revealed that PTPN21-upregulated BM-MSCs were insufficient in the immunosuppressive function, thereby limiting their clinical application.

The RNA-seq data showed that the downregulated DEGs were enriched in cell division and cell cycle, which further validated the above results of proliferation and cell cycle. Besides, the upregulated DEGs were enriched in cell chemotaxis. PTPN21-overexpressed BM-MSCs secreted more chemotactic factors such as *CXCL1*, *CXCL2*, *CXCL3*, and *α* chemokine interleukin-8 (*CXCL8*), which was consistent with previous reports that senescent cells secreted myriad factors, including growth factors, proteases, and cytokines [38, 43–45]. Chemokines have pleiotropic biological effects. They are generally known to regulate the recruitment and migration of leukocytes to sites of inflammation [46]. CXCL8 has been reported to promote tumor cell growth [47], induce migration of melanoma and breast carcinoma cells, and stimulate angiogenesis [48]. Moreover, BM-MSC-derived CXCL8 supported survival and proliferation of acute myeloid leukemia cells through the PI3K/AKT pathway [49]. Our migration assay showed that the expression level of PTPN21 did not affect the migration potential of BM-MSCs themselves, while the potential of PTPN21-overexpressed BM-MSCs recruiting human breast cancer cells and vascular endothelial cells was significantly higher than BM-MSC control cells. These data also suggested that overexpressed PTPN21 in BM-MSCs could promote bone marrow metastasis of tumor cells by inducing the secretion of multiple chemokines, but future studies of the detailed mechanisms are needed.

## 5. Conclusion

We have demonstrated for the first time that PTPN21 was involved in the regulation of biological characteristics of BM-MSCs. BM-MSCs overexpressing PTPN21 acquired a reduced proliferation level whereas gain higher differentiation potential including osteogenesis and adipogenesis. Additionally, PTPN21-upregulated BM-MSCs showed impaired immunosuppressive potential and accelerate recruitment of tumor cells and vascular endothelial cells. The results reveal the multiple roles of PTPN21 in BM-MSCs and suggest that the PTPN21 potentially become a new indicator of evaluating the quality and clinical availability of BM-MSCs.

## Figures and Tables

**Table 1:** Primers for RT-qPCR.

Genes	Primer sequence (5′ to 3′)
*GAPDH*	F:AGAAGGCTGGGGCTCATTTG
	R:AGGGGCCATCCACAGTCTTC
*PTPN21*	F:TTGGTTCCAGGCACAACACT
	R:TGTATTGGAGGTGCCAGACG
*SOX2*	F:ACTTTTGTCGGAGACGGAGA
	R:CATGAGCGTCTTGGTTTTCC
*Nanog*	F:TGCCTCACACGGAGACTGT
	R:CTTTGGGACTGGTGGAAGAAT
*OCT4*	F:GTATTCAGCCAAACGACCATCT
	R:GCTTCCTCCACCCACTTCT
*P21*	F:TGTCCGTCAGAACCCATGC
	R:AAAGTCGAAGTTCCATCGCTC
*P53*	F:CAGCACATGACGGAGGTTGT
	R:TCATCCAAATACTCCACACGC
*RUNX2*	F:CATAACCGTCTTCACAAATCC
	R:GTTCCCGAGGTCCATCTACT
*IBSP*	F:GGCAGTAGTGACTCATCCG
	R:AGCCCAGTGTTGTAGCAGA
*PPARγ*	F:ACCACTCCCACTCCTTTG
	R:GCAGGCTCCACTTTGATT
*FABP4*	F:GGATGATAAACTGGTGGTG
	R:GAACTTCAGTCCAGGTCAA
*FGF11*	F:CTGTACGCCTCTGCTCTCTAC
	R:GCCTTGGTCTTCTTAACTCGGT
*HSPA1A/B*	F:AGTCGGACATGAAGCACTGG
	R:TCTTGGTCAGCACCATGGAC
*HSPA2*	F:AGATCGACTCGCTCTACGAGG
	R:CGAAAGAGGTCGGCATTGAG
*HLA-DMB*	F:ACCTGTCTGTTGGATGATGCT
	R:CGCAAGGGGCCATCTTATTCT
*HLA-DRA*	F:TCTGGCGGCTTGAAGAATTTG
	R:GGTGATCGGAGTATAGTTGGAGC
*CCNB1*	F:AATAAGGCGAAGATCAACATGGC
	R:TTTGTTACCAATGTCCCCAAGAG
*TUBA4A*	F:TGAGATCCGAAATGGCCCATA
	R:TAGTGACCACGGGCATAGTTG
*FSCN1*	F:CACAGGCAAATACTGGACGGT
	R:CCACCTTGTTATAGTCGCAGAAC
*SNAI1*	F:TCGGAAGCCTAACTACAGCGA
	R:AGATGAGCATTGGCAGCGAG
*AURKB*	F:CGCAGAGAGATCGAAATCCAG
	R:AGATCCTCCTCCGGTCATAAAA
*BHLHE41*	F:TTAACCGCCTTAACCGAGCAA
	R:AGTGGAACGCATCCAAGTCG
*TUBB3*	F:GGCCAAGGGTCACTACACG
	R:GCAGTCGCAGTTTTCACACTC
*CHST4*	F:CCTCCCTCAACCTGCATATCG
	R:TCACAATGCGACTGTCAATCA
*EFNA4*	F:CTCCGCCACGTAGTCTACTG
	R:TACAAAGCAAACGTCTCGGGG
*FZD1*	F:ATCTTCTTGTCCGGCTGTTACA
	R:GTCCTCGGCGAACTTGTCATT
*GDF5*	F:TTCATCTGCACTGTGTTGGGT
	R:CCTGGCCTGAAGACGTTCC
*SFRP2*	F:AACCTACATCAACCGAGATACCA
	R:CTTCAGGTCCCTTTCGGACAC
*HLA-DQB1*	F:ACCTTCGGGTAGCAACTGTC
	R:AAATCCTCGGGAGAGTCTCTG
*CXCL1*	F:GCAGGGAATTCACCCCAAGA
	R:GATGCAGGATTGAGGCAAGC
*CXCL2*	F:GCTTGTCTCAACCCCGCATC
	R:TCCTCCTTCCTTCTGGTCAGT
*CXCL3*	F:CAGCGTATCATTGACACTTCCTG
	R:CCTTTCCAGCTGTCCCTAGAA
*CXCL8*	F:AAGGTGCAGTTTTGCCAAGG
	R:CCCAGTTTTCCTTGGGGTCC
*CXCL13*	F:GTGTGGACCCTCAAGCTGAA
	R:ACACTGGAACTGGTAGAGTTGA
*CCL20*	F:TATTGTGGGCTTCACACGGC
	R:GGATTTGCGCACACAGACAA
*CX3CL1*	F:TGCCCTAACTCGAAATGGCG
	R:GGCTCCAGGCTACTGCTTTC
*CXCR1*	F:CTGACCCAGAAGCGTCACTTG
	R:CCAGGACCTCATAGCAAACTG
*CXCR2*	F:CCTGTCTTACTTTTCCGAAGGAC
	R:TTGCTGTATTGTTGCCCATGT
*CXCR3*	F:CCACCTAGCTGTAGCAGACAC
	R:AGGGCTCCTGCGTAGAAGTT
*CXCR4*	F:ACGCCACCAACAGTCAGAG
	R:AGTCGGGAATAGTCAGCAGGA
*CXCR5*	F:CACGTTGCACCTTCTCCCAA
	R:GGAATCCCGCCACATGGTAG
*CCR6*	F:TTCAGCGATGTTTTCGACTCC
	R:GCAATCGGTACAAATAGCCTGG
*ATF4*	F:CCCTTCACCTTCTTACAACCTC
	R:TGCCCAGCTCTAAACTAAAGGA
*BIRC3*	F:TTTCCGTGGCTCTTATTCAAACT
	R:GCACAGTGGTAGGAACTTCTCAT
*ICAM1*	F:TTGGGCATAGAGACCCCGTT
	R:GCACATTGCTCAGTTCATACACC
*JAG1*	F:GTCCATGCAGAACGTGAACG
	R:GCGGGACTGATACTCCTTGA
*LIF*	F:CCAACGTGACGGACTTCCC
	R:TACACGACTATGCGGTACAGC
*CTH*	F:CATGAGTTGGTGAAGCGTCAG
	R:AGCTCTCGGCCAGAGTAAATA
*CBSL/CBS*	F:GGGTCCCCAGAGGATAAGGA
	R:GGGGTGTCCCCGATTTTCTT
*GOT1*	F:ATGGCACCTCCGTCAGTCT
	R:AGTCATCCGTGCGATATGCTC
*PSAT1*	F:TGCCGCACTCAGTGTTGTTAG
	R:GCAATTCCCGCACAAGATTCT
*FABP4_mouse_*	F:AACACCGAGATTTCCTT
	R:ACACATTCCACCACCAG
*PPARγ_mouse_*	F:TCGCTGATGCACTGCCTATG
	R:GAGAGGTCCACAGAGCTGATT
*β-actin_mouse_*	F:GGCTGTATTCCCCTCCATCG
	R:CCAGTTGGTAACAATGCCATGT

## Data Availability

The RNA-seq data used in our study have been deposited in NCBI's SRA database and are accessible through the accession number PRJNA525776 [50].

## Abbreviations

PTPN21: Protein tyrosine phosphatase nonreceptor type 21
MSCs: Mesenchymal stem cells
BM-MSCs: Bone marrow-derived mesenchymal stem cells
GVHD: Graf-versus-host disease
PCR: Polymerase chain reaction
DEGs: Differentially expressed genes
ALL: Acute lymphoblastic leukemia
allo-HSCT: Allogeneic hematopoietic stem cell transplantation
ECs: Human vascular endothelial cells.

## Supplementary Materials

Supplementary Materials Supplemental figure 1: KEGG pathway enrichment analysis of RNA-seq data. The left and right column charts showed the terms of ranking top five downregulated pathways and upregulated pathways, respectively. Supplemental figure 2: identification of RNA-seq results by RT-qPCR and western blot assays. Supplemental figure 3: statistical analysis of adipogenic differentiation data of transfected or untransfected 3T3-L1 cells. (a-b) Statistical analysis of mRNA expression of the adipogenic markers FABP4 and PPAR-*γ* at day 5 and day 10 of induction in two groups of transfected 3T3-L1 cells. (c) mRNA expression of PPAR-*γ*, FABP4, and PTPN21 during adipogenic differentiation of untransfected 3T3-L1 cells.
