# Supplementary material for: PTPN21 Overexpression Promotes Osteogenic and Adipogenic Differentiation of Bone Marrow-Derived Mesenchymal Stem Cells but Inhibits the Immunosuppressive Function
Source: Stem Cells Int. 2019 Nov 21;2019:4686132. doi: 10.1155/2019/4686132 (PMC6907062; doi:10.1155/2019/4686132)
Supplement: Supplementary Materials — Supplemental figure 1: KEGG pathway enrichment analysis of RNA-seq data. The left and right column charts showed the terms of ranking top five downregulated pathways and upregulated pathways, respectively. Supplemental figure 2: identification of RNA-seq results by RT-qPCR and western blot assays. Supplemental figure 3: statistical analysis of adipogenic differentiation data of transfected or untransfected 3T3-L1 cells. (a-b) Statistical analysis of mRNA expression of the adipogenic markers FABP4 and PPAR-γ at day 5 and day 10 of induction in two groups of transfected 3T3-L1 cells. (c) mRNA expression of PPAR-γ, FABP4, and PTPN21 during adipogenic differentiation of untransfected 3T3-L1 cells. [file 4686132.f1.zip › Summary of plasmid profiles.pdf]

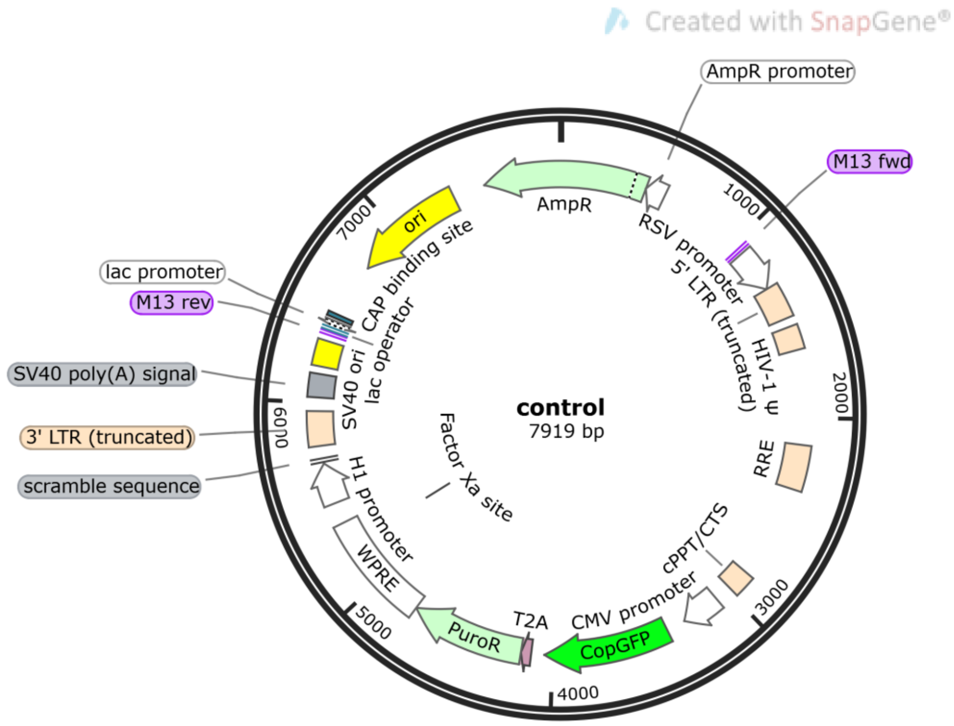


Figure 1. Lentiviral control plasmid

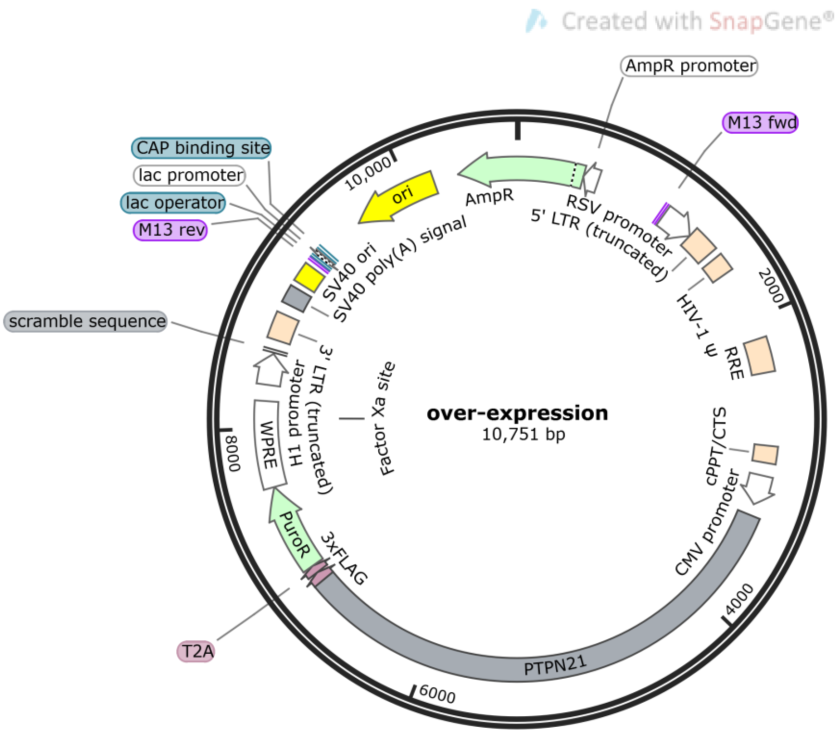


Figure 2. Lentiviral PTPN21 overexpression plasmid

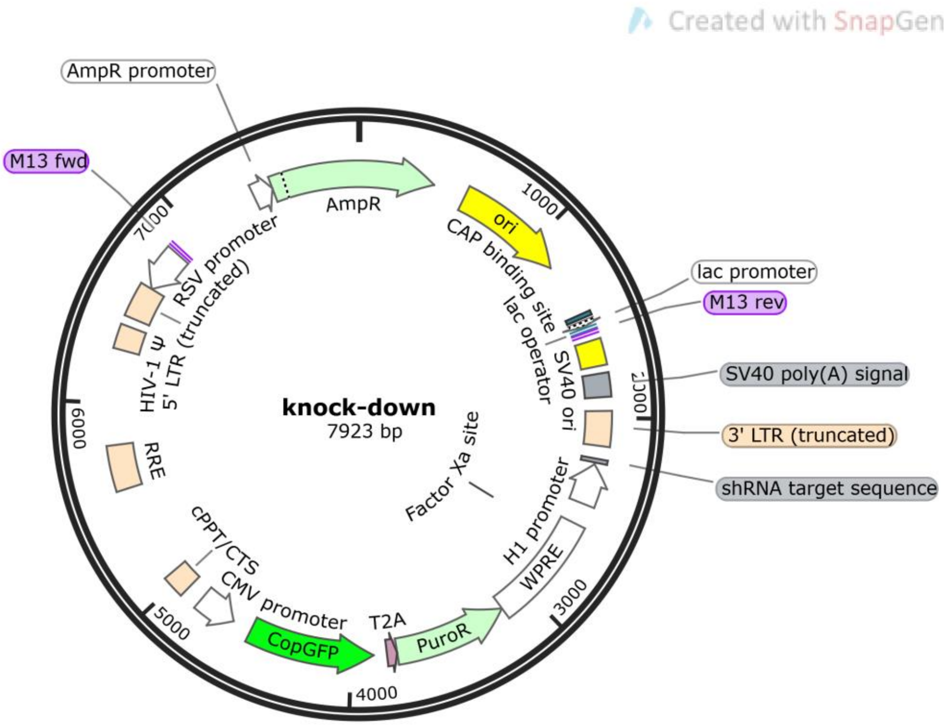


Figure 3. Lentiviral PTPN21 knock-down plasmid
